# The Transthyretin/Oleuropein Aglycone Complex: A New Tool against TTR Amyloidosis

**DOI:** 10.3390/ph15030277

**Published:** 2022-02-23

**Authors:** Francesco Bemporad, Manuela Leri, Matteo Ramazzotti, Massimo Stefani, Monica Bucciantini

**Affiliations:** Department of Experimental and Clinical Biomedical Sciences “Mario Serio”, University of Florence, Viale Morgagni 50, 50134 Florence, Italy; francesco.bemporad@unifi.it (F.B.); matteo.ramazzotti@unifi.it (M.R.); massimo.stefani@unifi.it (M.S.); monica.bucciantini@unifi.it (M.B.)

**Keywords:** transthyretin, polyphenols, systemic amyloidosis, misfolding

## Abstract

The release of monomers from the homotetrameric protein transthyretin (TTR) is the first event of a cascade, eventually leading to sporadic or familial TTR amyloidoses. Thus, ligands able to stabilize TTR and inhibit monomer release are subject of intense scrutiny as potential treatments against these pathologies. Here, we investigated the interaction between TTR and a non-glycated derivative of the main olive polyphenol, oleuropein (OleA), known to interfere with TTR aggregation. We coupled fluorescence studies with molecular docking to investigate the OleA/TTR interaction using wild-type TTR, a monomeric variant, and the L55P cardiotoxic mutant. We characterized a fluorescence band emitted by OleA upon formation of the OleA/TTR complex. Exploiting this signal, we found that a poorly specific non-stoichiometric interaction occurs on the surface of the protein and a more specific stabilizing interaction takes place in the ligand binding pocket of TTR, exhibiting a *K_D_* of 3.23 ± 0.32 µM, with two distinct binding sites. OleA interacts with TTR in different modes, stabilizing it and preventing its dissociation into monomers, with subsequent misfolding. This result paves the way to the possible use of OleA to prevent degenerative diseases associated with TTR misfolding.

## 1. Introduction

Transthyretin (TTR), also known as prealbumin, is a 55 KDa circulating protein produced by liver and, to a lesser extent, by choroid plexus, with a well-established role in the transport of thyroxine (T4) and retinol in the plasma and the cerebrospinal fluid [1]. Structurally, TTR is a homotetramer, where the four monomers are assembled with a 2:2:2 symmetry resulting in a dimer of dimers. In each dimer, monomer interaction is stabilized through H-bonds involving the two-edge H and F β-strands; the back-to-back association of the two dimers is stabilized by a limited number of contacts, giving rise to two symmetrical binding sites for T4, at the dimer–dimer interface [2]. According to computational analysis, human TTR possesses a relatively high intrinsic propensity to β-aggregation [3], and such a propensity is strongly enhanced in a set of genetic TTR variants as a result of a destabilization of the native TTR structure [4].

Presently, over 130 TTR gene mutations have been associated with pathological phenotypes, with autosomal dominant inheritance [5,6,7,8]. Under proper conditions, wild-type TTR (wt-TTR) or TTR variants polymerize into amyloid fibrils in vivo, similarly to over 30 other known peptides/proteins involved in different amyloid diseases. Most of the reported TTR mutations result in loss of tetramer stability, with tetramer dissociation into misfolded monomers and dimers that undergo aberrant partial refolding with generation of amyloidogenic intermediates [9]. Importantly, a strong correlation does exist between the thermodynamic/kinetic stability of TTR variants and their propensity to grow into misfolded, soluble and insoluble aggregates [10,11]. Considering the importance of TTR tetramer stability and the structure of the monomer, an engineered monomeric variant of TTR (M-TTR) has been generated. M-TTR is a F87M/L110M double mutant rationally designed to modify the monomer–monomer interface, such that tetramer formation under physiological conditions is hindered [12]. 

The different forms of senile or familial systemic TTR amyloidosis (A-TTR) are characterized by the presence, in the affected tissues/organs, of extracellular deposits of amyloid fibrils composed of TTR polymers. A-TTR include senile systemic amyloidosis (SSA), familial amyloid cardiomyopathy (FAC), familial amyloid polyneuropathy (FAP), and central nervous system selective amyloidosis (CNSA). SSA is a common, age-related, amyloidosis characterized by the accumulation of wt-TTR fibrils [1], predominantly in the heart and, to a lesser extent, in other organs [13,14]. Depending on the type of A-TTR, the clinical features of these pathologies include a diffuse polyneuropathy, a severe form of cardiomyopathy, or severe neurological symptoms; the latter include seizures, stroke-like episodes, dementia, psychomotor deterioration, hydrocephalus, spinal cord infarction, and variable amyloid deposition in the vitreous humor. In addition to the peripheral nerves and the myocardium, pathological amyloid deposits of TTR are detected in the walls of leptomeningeal vessels and the surrounding connective tissue structures, as well as in the pia arachnoid and subpial region, with risk of cerebral infarction and, in later stages, cerebral hemorrhage [5,15]. In particular, a severe form of FAP, characterized by early-onset and rapid progressive amyloidosis with severe cardiac involvement (FAC), results from amyloid deposits in the heart parenchyma of a number of mutants, particularly the highly amyloidogenic Leu55Pro (L55P) mutant [16,17,18].

A-TTR are severe diseases due to the substantial lack of effective therapies other than symptomatic ones, and also since their progression is already in an advanced stage when their signs and symptoms appear. In addition, the increased aging of the population potentially fosters the cases of amyloidosis involving wt-TTR (SSA). Accordingly, if coupled to the pharmacological tools presently available, early diagnosis and prevention are important to slow disease progression and to improve its prognosis.

Recently, different approaches targeting amyloid deposits and interfering with organ damage have been developed that are being tested in clinical trials (see later). The results of these ongoing trials, together with a better understanding of the amyloidogenic process and an improved knowledge of the mechanisms of organ damage, will help to establish the role of novel agents and to identify potential alternative therapeutic targets.

The usefulness of exploiting stabilizers of the tetrameric structure of TTR avoiding its disassembly has led us, in recent years, to develop molecules, such as diflunisal and Tafamidis, that associate with and stabilize TTR by occupying the central cavity of the tetramer normally designed to harbor T4. Preclinical studies and clinical trials have supported the protection by these molecules against disease occurrence [19]. Diflunisal is an off-label treatment option whose use has limitations due to its non-steroidal anti-inflammatory drug (NSAID) properties [20]. Tafamidis stabilizes both wt-TTR and 14 TTR variants clinically tested, as well as 25 TTR variants tested ex vivo [21]. Presently, Tafamidis is the first Food and Drug Administration (FDA) and European Medicines Agency (EMA)-approved targeted treatment for TTR cardiomyopathy (ATTR-CM). More recently, other molecules have been developed; these include AG10, a small molecule TTR stabilizer [22], and PRX004, the first investigated intravenous immunotherapy drug for treatment of A-TTR (https://clinicaltrials.gov/ct2/show/NCT03336580; accessed on 20 January 2022). Patisiran and Inotersen are silencers of TTR mRNA that have been approved by FDA for TTR polyneuropathy. These molecules work to prevent translation and to reduce TTR production by hepatocytes [23]. Patisiran is a small interfering RNA (siRNA) that targets the 3′ untranslated region of TTR mRNA, causing TTR mRNA degradation through RNA interference [24]. Inotersen reduces TTR production by binding to TTR mRNA, with ensuing reduction in serum TTR levels and TTR deposits in tissues (https://www.ema.europa.eu/en/medicines/human/EPAR/tegsedi; accessed on 20 January 2022). Other molecules able to interfere with TTR production, unfolding, and aggregation presently under investigation include TTR fibril disrupters such as doxycycline, which target amyloid deposits to prevent further tissue damage [23].

In this context, several natural polyphenols have been reported to interfere with the misfolding of several amyloidogenic proteins and to inhibit their aggregation into amyloid. In particular, attention has been focused on some flavonoids [25] and natural polyphenols in relation to their ability to bind and to stabilize TTR, hindering its fibrillization. The most investigated plant polyphenols include resveratrol [26], curcumin [27], epigallocatechin 3-gallate (EGCG) [28], and more recently, oleuropein aglycone (OleA), the main phenolic component of the extra virgin olive oil [29]. Notably, an EGCG preparation (GTE) is a nonpharmacological option that is used for the treatment of ATTR-CM [23].

Most of the studies reported above have shown that all these compounds stabilize TTR and inhibit its aggregation by strongly suppressing tetramer dissociation, maintaining protein solubility [28,29], or alternatively, by inducing TTR oligomerization into a homogeneous population of small off-pathway non-toxic aggregates [30]. We previously reported that OleA does not inhibit aggregation of both wt-TTR and L55P-TTR; rather, it reduces the exposure of protein aromatic residues during the amyloidogenic process. We found that the presence of OleA during non-native aggregation under acid conditions of wt-TTR and L55P-TTR induced an increase in Trp quenching; this finding suggests that the stabilization by OleA of meta-stable misfolded intermediates of TTR relies on the reduction in their surface hydrophobicity. Overall, the data reported suggest that OleA induces some remodeling of the supramolecular structure of the growing aggregates [29].

Although our previous work highlighted the ability to alter aggregation exerted by OleA, those data were collected under non-physiological acidic conditions. Consequently, in the present study, we further investigated the TTR-OleA relation by analyzing in depth the native interaction between OleA and different forms of TTR (wt-TTR, L55P-TTR and M-TTR) under physiological conditions to improve the knowledge, at a molecular level, of both the anti-amyloidogenic activity of this polyphenol and the underlying molecular mechanisms. These aspects are of great importance to assess the possible use of this plant polyphenols as natural TTR stabilizers to prevent the onset of different sporadic and familial TTR amyloidosis. Indeed, a deeper knowledge of the OleA-TTR interaction is a prerequisite to devise new therapeutic strategies aimed at exploiting these natural compounds, alone or in combination with other molecular treatments, to prevent A-TTR and other amyloid-associated diseases.

## 2. Results

### 2.1. Molecular Docking of Polyphenols to Native TTR

**Figure 1:**
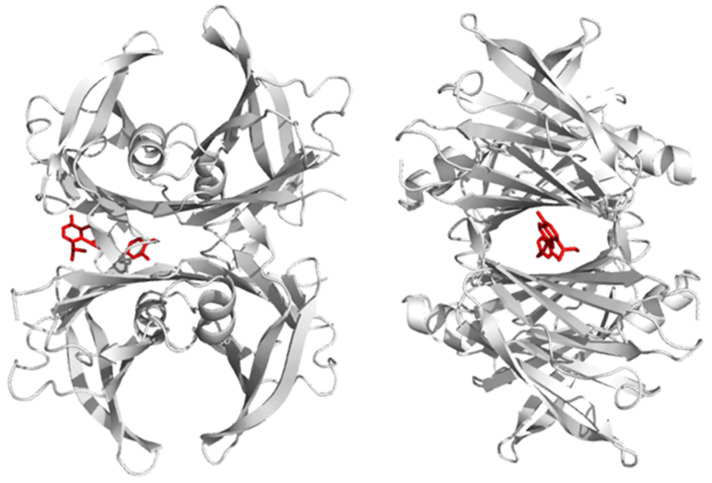
Orthogonal side views of the TTR tetramer binding OleA in the best (most stable) pose, as determined by molecular docking experiments performed with Autodock-Vina.

We carried out molecular docking experiments to explore the possible ability of small molecules, namely Oleuropein (Ole), Oleuropein aglycone (OleA), T4, and resveratrol, to bind the crystal structure of the biologically active wt-TTR and its mutant L55P-TTR. Autodock-Vina was asked to suggest the 20 best poses showing a difference of at most 3 Kcal/mol from the best and the worst pose. In a free-to-bind (i.e., not indicating a specific docking area) experiment, T4 was correctly located into the already-described C2 symmetric funnel-shaped T4 binding site at the dimer–dimer interface (Figure 1), with a binding energy of −5.9 Kcal/mol. The interaction of resveratrol in the same site was even stronger (−6.3 Kcal/mol). On the contrary, the best pose of Ole was not in the main funnel; rather, it was localized in the secondary funnel, identified as the halogen binding site [31]. However, removal of the sugar moiety from Ole led OleA to interact with the main funnel very strongly (−7.7 Kcal/mol), thus displaying the highest affinity in this experimental set-up.

We then repeated the docking simulation by specifically testing the penetration into, and the binding to, the main funnel of the investigated molecules. T4 penetrated deeper in the funnel than in the previous setup, although with a lower interaction energy (−5.0 Kcal/mol). The opposite behavior was observed when studying resveratrol, which penetrated less deeply but with a stronger interaction energy (−7.6 Kcal/mol). Ole interaction in the tested main funnel was deep and with a docking energy slightly lower than resveratrol (−7.4 Kcal/mol). Again, OleA exhibited the highest interaction energy (−8.1 Kcal/mol).

Given the cooperative nature of T4 binding to TTR, we also checked whether the previously tested, ligand-free TTR was not sufficiently prepared to host ligands (considering that, during docking, the involved part of the protein is considered as a rigid body). Therefore, we repeated the experiments with a different crystal structure of TTR, where the main funnel was originally bound to T4, allowing the protein to be considered in an “open” state. We prepared this new experiment by removing T4 from the main funnel and repeated the analysis as before. This time, all tested molecules spontaneously (i.e., without targeting) docked into the main funnel, and OleA displayed the highest affinity (−8.8 Kcal/mol), followed by resveratrol (−6.9 Kcal/mol), T4 (−6.3 Kcal/mol), and Ole (−6.0 Kcal/mol). Thus, when tested in silico, OleA exhibits an affinity for the TTR binding pocket comparable to, if not higher than, the affinities displayed by other known ligands.

### 2.2. Excitation−Emission Matrices

**Figure 2:**
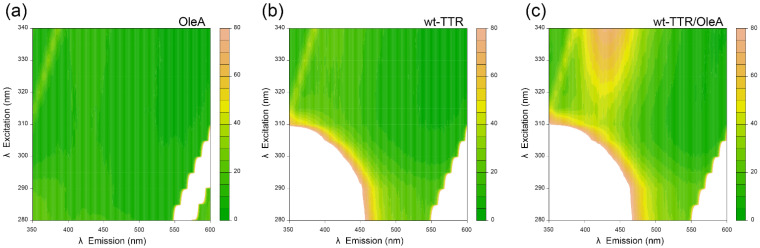
Excitation−emission matrices (EEM) in the 280–340 nm excitation range and in the 350--600 nm emission range. The samples tested were (**a**) OleA, (**b**) wt-TTR, and (**c**) the complex wt-TTR/OleA incubated at pH 7.0 and 37 °C for 24 h. In all matrices, the white-colored band on the bottom right corner corresponds to the harmonic (double wavelength) of the incoming light, which gives an intense signal that reaches the top-scale; the small yet detectable signal in the top left corner (emission–excitation coordinates going from 350–315 to 400–340 nm) corresponds to the Raman scattering of water.

We then investigated the OleA-wt-TTR interaction in vitro by recording fluorescence emission–excitation matrices, using fluorescence wavelengths in the 280–340 nm range for excitation and in the 350–600 nm range for emission. Figure 2 shows that the excitation–emission matrix of OleA alone did not reveal detectable fluorescence emission; the matrix obtained for wt-TTR displayed the intrinsic fluorescence typical of a buried tryptophan upon excitation in the 280–310 nm range, namely a band centered at 335 nm and no emission at higher wavelengths (Figure 2b). Interestingly, we observed a different behavior when OleA and TTR were incubated together for 24 h at a concentration of 15 µM and 5.0 µM, respectively. In this case, a previously undetected signal appeared, located between 400 and 450 nm. This signal could not be observed when OleA or TTR were present alone in solution, making this band a possible candidate to investigate the interaction between these two species.

### 2.3. Investigation of the Interaction between OleA and wt-TTR

**Figure 3:**
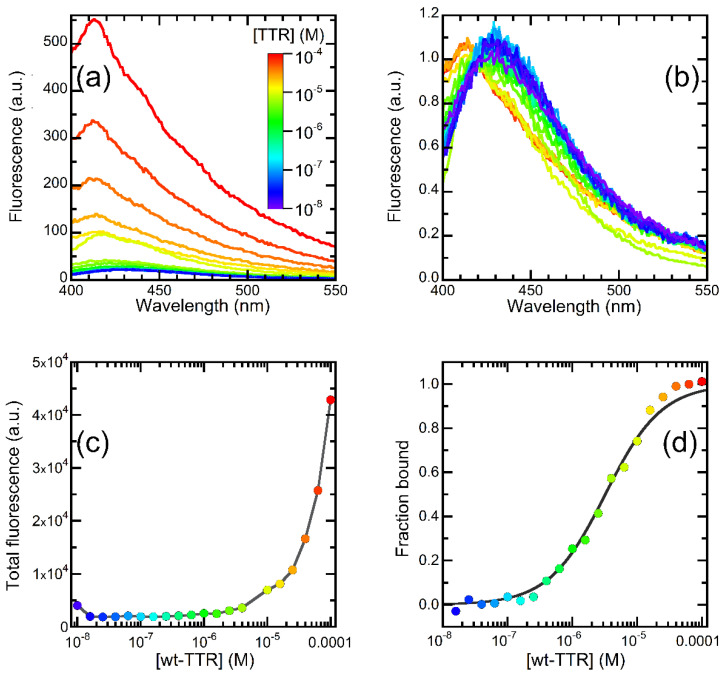
OleA-wt-TTR interaction. (**a**) Fluorescence emission spectra of OleA in the presence of increasing concentrations of wt-TTR in the 1 × 10^−8^ M-10^−4^ M range. The color varies from blue to purple as the protein concentration increases from 1 × 10^−8^ M to 1 × 10^−4^ M, as shown in the color scale. (**b**) The same spectra shown in panel (**a**), normalized to the signal emitted at 420 nm to highlight the blue shift of the emission we observed on increasing wt-TTR concentration. Color scale as in panel (**a**). (**c**) Total emission intensities of the spectra reported in panel (**a**) plotted vs. wt-TTR concentration. The concentration refers to the tetramer. Color scale as in panel (**a**). (**d**) The fraction of wt-TTR bound to OleA plotted vs. wt-TTR concentration. The concentration refers to the tetramer. We calculated the fraction bound from the analysis of the blue shift of the emission, as reported in the Methods section. Color scale as in panel (**a**).

Following our initial observation of a so far undetected fluorescence band in the 400–450 nm range emitted by OleA in the presence of TTR, we sought to exploit this signal to extract quantitative information about the interaction between the two molecules. In a first experiment, we evaluated the fluorescence measured at a constant concentration of OleA, in the presence of increasing concentrations of wt-TTR. The fluorescence spectra, reported in Figure 3a, indicate that the fluorescence intensity of OleA was substantially unaffected at protein concentrations below 0.1 µM (Figure 3a), whereas at protein concentrations >0.1 µM, the signal displayed two effects; first, the signal increased as the wt-TTR concentration increased (Figure 3a). The total fluorescence emitted by OleA in the presence of 100 µM wt-TTR was 10.6 ± 1.5 times higher than the signal emitted in the presence of 10 nM wt-TTR. Second, the signal displayed a blue shift that was more evident as the concentration of wt-TTR increased. The peak wavelengths of OleA were 435 or 413 nm in the presence of 10 nM or 100 µM wt-TTR, respectively. To highlight this effect, we normalized the spectra to the signal emitted at 420 nm (Figure 3b).

To extract a dissociation constant *(K_D_*) value for the OleA-TTR complex from the fluorescence data, we plotted total OleA fluorescence vs. wt-TTR concentration (Figure 3c). The results of this analysis confirmed the increase in the signal observed on increasing wt-TTR concentration. However, the trace obtained did not reach a plateau, even at wt-TTR concentrations in the 10–100 µM range, the maximum protein concentration we could reach in our experimental setting. Consequently, we could not extract a quantitative parameter from these data. When we considered the emission wavelength, a different trend was observed. In fact, the fluorescence spectra began to exhibit a significant blue shift at wt-TTR concentrations above 0.1 µM, and this shift reached a plateau starting from 40 µM. We could observe a plateau by plotting either the center of mass (COM) or the ratio between the fluorescence at 428 and 413 nm (Supplementary Figure S1). This trend is suggestive of a binding process that begins at protein concentrations above 0.1 µM and reaches saturation at protein concentrations above 40 µM. Consequently, the data reported in Figure 3d were normalized to the bound fraction, i.e., the fraction of OleA bound to wt-TTR under a certain condition. We plotted the bound fraction vs. protein concentration (Figure 3d) and fitted the data to a binding equilibrium, as reported in the Methods section. The outcome of this analysis yielded a *K*_D_ value of 3.23 ± 0.32 µM. Thus, our data show that it is possible to exploit the fluorescence emitted by OleA in the presence of wt-TTR to gauge affinity parameters.

### 2.4. Investigation of the Interaction between OleA and TTR Mutants

**Figure 4:**
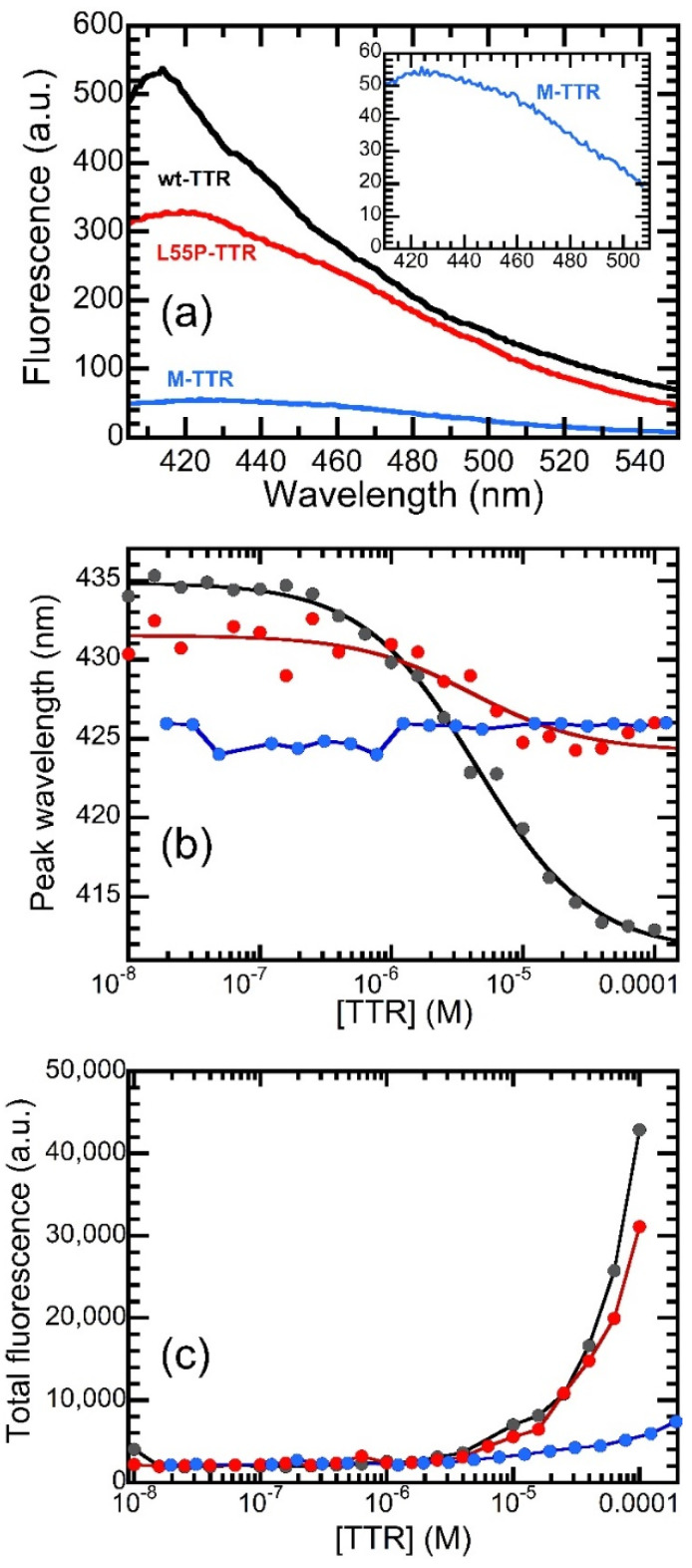
Interaction between OleA and TTR mutants. (**a**) Comparison between the emission of OleA at a concentration of 0.5 µM in the presence of 100 µM wt-TTR (black), L55P-TTR (red), and M-TTR (blue). The inset shows a magnification of the spectrum recorded in the presence of M-TTR. (**b**) Peak wavelength of the OleA spectra as a function of the concentration of wt-TTR (black), L55P-TTR (red), and M-TTR (blue). (**c**) Total fluorescence emitted by OleA as a function of the concentration of wt-TTR (black), L55P-TTR (red), and M-TTR (blue).

For comparison with the data obtained with wt-TTR, the same experiment was repeated in the presence of two TTR mutants, L55P-TTR and M-TTR. Indeed, the binding pocket is known to be altered in the L55P variant and absent in M-TTR. The emission spectra of 0.5 µM OleA recorded in the presence of different concentrations of the two variants are shown in Supplementary Figure S2a, where the spectra measured in the presence of the two variants at 100 µM protein concentration are compared with that of wt-TTR. These spectra illustrate that the two mutant proteins induced a different effect when compared to wt-TTR; in fact, the blue shift shown by OleA in the presence of L55P-TTR was much less intense than that observed with wt-TTR (Figure 4a). Indeed, the emission peak shifted from 431.2 nm to 425.2 nm upon increasing L55P-TTR concentration from 0.01 to 100 µM, a 6.0 nm shift that was much lower than the 22 nm shift observed with wt-TTR (Figure 4b). Although the reduced shift observed made the data noisier, we attempted a quantitative analysis to calculate the OleA-L55P-TTR affinity constant, as reported above for wt-TTR. The analysis yielded a *K_D_* value of 3.12 ± 0.62 µM, which is, within the experimental error, substantially identical to the affinity calculated for wt-TTR. When the M-TTR data were analyzed, we found that this variant was unable to induce any significant shift of OleA emission. In fact, even at low protein concentration, the emission of OleA occurred at slightly shorter wavelengths that with wt-TTR: the peak was present at 426 nm even in the presence of M-TTR concentrations as low as 0.01 µM. However, the peak remained in the same position even when the protein concentration was increased up to values of 195 µM, the maximum concentration we could reach.

A different trend was observed when the emission intensity was taken into consideration (Supplementary Figure S2 and Figure 4c). In fact, both mutants were able to induce an increase in the emission of OleA, yet with different intensities. The total emission values in the presence of 100 µM protein were 42,800, 31,100, and 5600 a.u. for wt-TTR, L55P-TTR, and M-TTR, respectively. However, it is important to point out that the concentrations shown in Figure 4c are molar concentrations. Given the monomeric nature of M-TTR, the different ability of OleA to induce fluorescence increases may be at least partially compensated for by normalizing the data to the mass concentration. This mathematical operation would reduce the emission intensities of the tetrameric wt-TTR and L55P-TTR by four times.

### 2.5. Biophysical Characterization of the Emission by OleA

**Figure 5:**
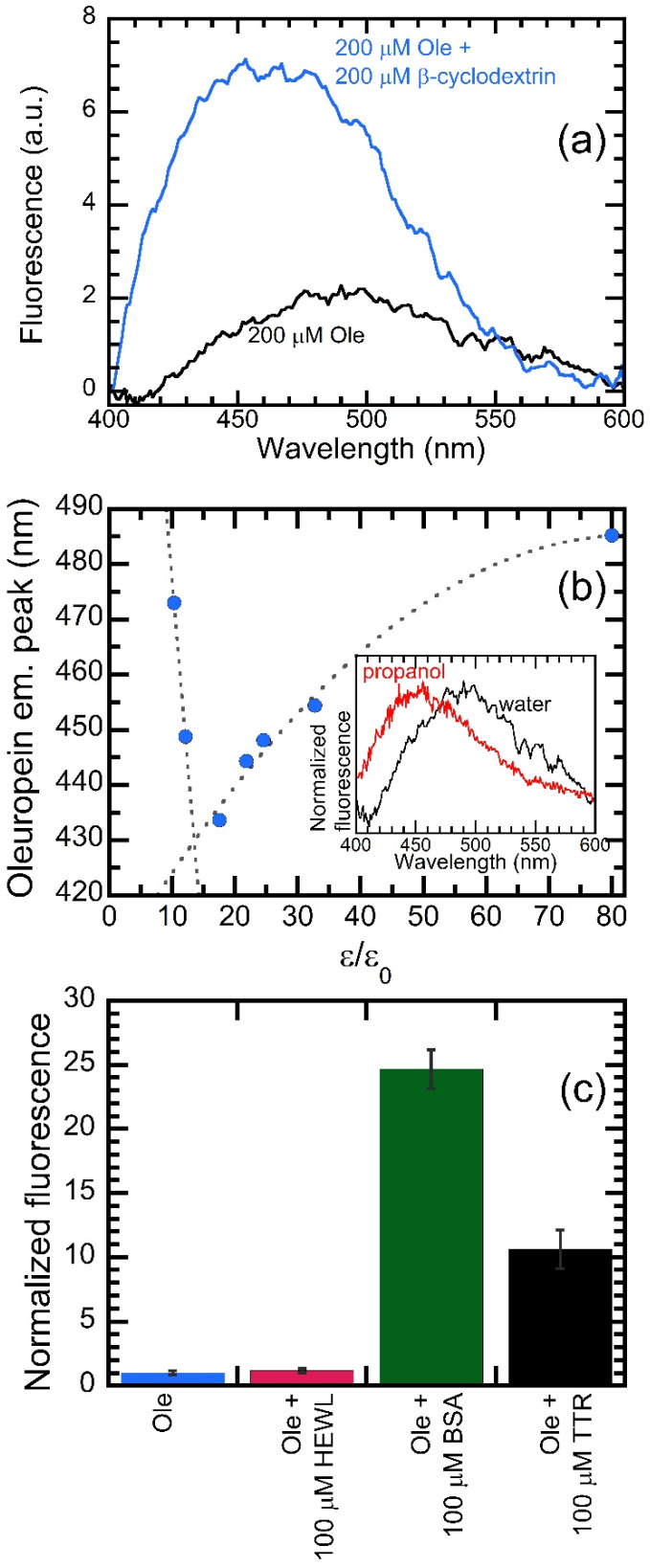
Biophysical characterization of the fluorescence emitted by OleA. (**a**) Fluorescence spectra of 200 µM OleA recorded in water in the absence (black) or in the presence (blue) of 200 µM β-cyclodextrin. (**b**) OleA emission peak as a function of solvent relative permittivity (ε/ε_0_). The inset shows a comparison between the OleA emission spectra recorded in water (ε/ε_0_ = 79.4) (black) or in propanol (ε/ε_0_ = 21.8) (red). (**c**) Comparison of total OleA fluorescence intensity measured in the presence of 100 µM hen egg white lysozyme (HEWL), bovine serum albumin (BSA), or wt-TTR.

The change in OleA fluorescence we observed remains elusive and cumbersome to interpret. The question arises as to whether the observed changes of emission correspond to a true binding event and whether or not this binding occurs within the binding site of TTR, or rather, it results from a non-specific interaction at the protein surface. Therefore, to better interpret our data, we made a series of measurements of OleA fluorescence. First, we investigated OleA emission in the presence of β-cyclodextrin (βCD), a cyclic oligosaccharide containing seven glucose molecules with a hydrophilic outer surface and a hydrophobic inner cavity, known to be able to harbor phytochemicals [32]. We measured the emission of OleA in water, in the absence or in the presence of an identical molar concentration of βCD (Figure 5a). We found that the emission of OleA displayed increased intensity and a blue shift in the presence of βCD. In fact, the total fluorescence emitted in the presence of βCD was 3.3 ± 0.1 times higher than that measured in the absence of the molecule. As far as the emission wavelength is concerned, we measured peak wavelengths of 459 and 485.2 nm in the presence and in the absence of βCD, respectively.

Next, to possibly assign the emission of OleA to the effect of a hydrophobic chemical environment, we measured the fluorescence spectra of OleA in different solvents with varying relative permittivity (ε/ε_0_) (Figure 5b). When we plotted the peak wavelength vs. ε/ε_0_, a significant blue shift was observed upon increasing the hydrophobicity. In fact, in water (ε/ε_0_ = 80) the peak was present at 485.2 nm, whereas in 1-buthanol (ε/ε_0_ = 17.5), the peak was located at 433.7 nm, leading us to establish a direct link between the hydrophobicity of the chemical environment and the observed blue shift.

We also investigated whether the ability to induce an increase in OleA fluorescence was a specific feature of TTR or, rather, it was shared with other proteins. A series of samples containing OleA in the presence of the same concentration (100 µM) of different proteins, hen egg white lysozyme (HEWL), bovine serum albumin (BSA) and wt-TTR were investigated. The fluorescence intensities at 485 nm of these samples were measured and normalized at the value observed in the absence of proteins (Figure 5b). The results of this experiment are rather complex to elucidate. HEWL did not induce any significant increase in fluorescence intensity of OleA. The signal in the presence of 100 µM HEWL was 1.16 ± 0.17 times higher than that measured in the absence of protein. However, in the presence of BSA, we observed a completely different effect: the signal was 24.7 ± 1.5 times higher than that measured for OleA alone. This increase was greater than that recorded in the presence of an identical amount of wt-TTR. Indeed, we observed that the signal in the presence of 100 µM wt-TTR was 10.6 ± 1.5 times higher than that measured without the protein. We conclude that the ability to induce an increase in OleA fluorescence is not a specific feature of TTR; rather, it is a property shared by TTR with other, but not all, proteins. Probably, the increased effect we observed depends on surface properties —mainly in terms of hydrophobicity—of the investigated protein.

**Figure 6:**
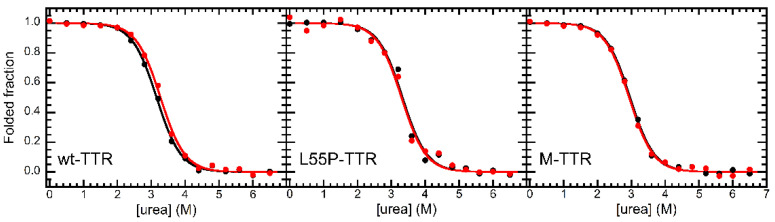
Equilibrium unfolding experiments of TTR in the absence (black) and in the presence (red) of 100 µM OleA. Data reported refer to wt-TTR (**left**), L55P-TTR (**center**), and M-TTR (**right**). Continuous lines represent best fits of data to the model edited by Santoro and Bolen [33].

Finally, we investigated the effect of OleA binding on the thermodynamic stability of TTR by means of urea-induced equilibrium unfolding experiments in the presence and in the absence of 100 µM OleA (Figure 6). The tryptophan fluorescence spectra were collected and the data were analyzed as reported in the Methods section. The results of this analysis indicate that, in the case of wt-TTR, OleA binding induced a slight, yet significant, increase in the thermodynamic stability of the protein. The free energy change upon unfolding ($\Delta G_{U-F}^{H_{2}O}$) were 22,600 ± 200 and 21,800 ± 200 J/mol in the presence and in the absence of OleA, respectively. Different results were obtained with L55P-TTR and M-TTR. The values of $\Delta G_{U-F}^{H_{2}O}$ obtained for L55P-TTR were 23,000 ± 300 and 22,800 ± 300 J/mol in the absence and in the presence of the ligand, respectively, whereas the values of $\Delta G_{U-F}^{H_{2}O}$ obtained for M-TTR were of 20,400 ± 200 and 20,200 ± 200 J/mol in the absence and in the presence of the ligand, respectively. This experiment further supports the pieces of evidence concerning TTR-OleA interaction. Ligand binding to the wild-type protein induces an increase in the thermodynamic stability of the tetramer. However, L55P-TTR has a compromised binding pocket that alters the binding, leaving protein stability unaffected by the interaction. We also found that M-TTR is unable to bind OleA and, accordingly, we could not detect any significant effect on the thermodynamic stability of the protein in the presence of the ligand.

### 2.6. Effects of Oleuropein Aglycone on TTR under Physiological Conditions

**Figure 7:**
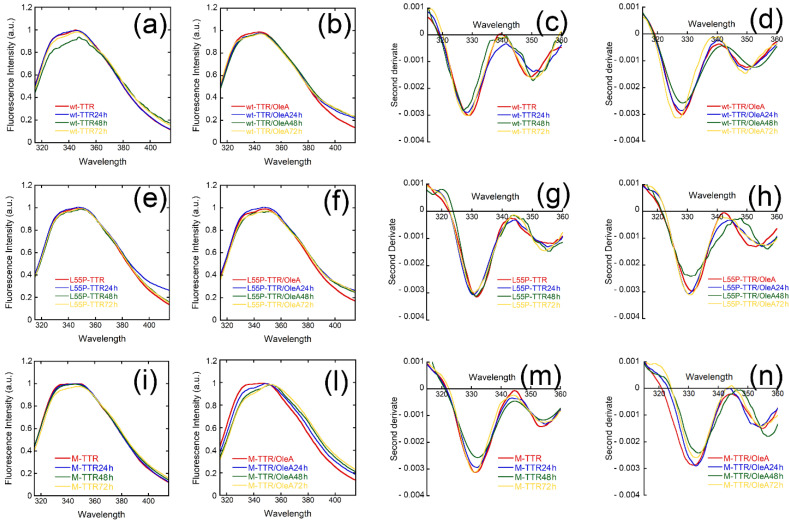
Intrinsic fluorescence emission spectra recorded at different times of incubation of wt-TTR (**a**), L55P-TTR (**e**), and M-TTR (**i**) before and after OleA supplementation to the incubation mixture (**b**,**f**,**l**). The spectra were normalized to fluorescence intensity of 1.0 at λ_max_, λ_exc_ = 295 nm. (**c**,**d**,**g**,**h**,**m**,**n**). The second derivatives were obtained from the previous emission spectra.

Our previous data showed that OleA interacts specifically in the tryptophan (Trp) region of TTR during the aggregation process, under acidic conditions [29]. In the present study, we analyzed the effect of OleA on the native tetrameric structure of wt-TTR, L55P-TTR, and M-TTR under physiological conditions (pH 7.0). The data obtained by the equilibrium unfolding were confirmed by the analysis of TTR intrinsic fluorescence in the presence or in the absence of OleA at different incubation times (Figure 7). Second derivate spectra showed two peaks at around 330 nm and 350 nm that are associated with different polarities of the environment surrounding aromatic residues due to the presence, in each TTR monomer, of two Trp residues at positions 41 and 79. The 330 nm peak, typical of the natively folded protein with buried indole moieties, is the most evident, whereas the less intense 350 nm peak is due to the contribution of the solvent-exposed Trp. In our experimental setting, we found that the two peaks were unaffected by the presence of OleA, also after protein incubation for 72 h, confirming that the OleA/TTR interaction does not affect the structure of the native protein.

## 3. Discussion

The knowledge of the mechanism by which polyphenolic compounds interfere with amyloid aggregation is of great importance, considering that (i) the growth of amyloid assemblies is a common feature of many systemic and neurodegenerative diseases, including type 2 diabetes, FAP, FAC, AL-amyloidosis, Alzheimer’s disease, Parkinson’s disease, Creutzfeldt–Jakob’s disease, Huntington’s disease, and many others; (ii) a large number of studies indicate that these molecules are able to interfere in several ways with protein misfolding and the ensuing growth of amyloid assemblies; and (iii) the selection and development of small molecules able to inhibit, or to interfere with, the growth of toxic protein assemblies, most often unstable pre-fibrillar aggregates, or to reduce their ability to bind to the cell membrane could have a considerable clinical application. Therefore, establishing the mechanism by which polyphenols disrupt aggregation-prone conformations is of paramount importance to reach a complete knowledge of the structure–activity relationships of these natural compounds. Recent papers suggest that aromatic interactions favor molecular recognition of amyloidogenic sequences by enhancing the directionality and orientation needed for the ordered self-assembly process and hence fibril assembly kinetics [34].

It has been reported that several polyphenols interact with amyloidogenic aromatic residues hindering the stacking of π-systems [35,36], thus inhibiting the elongation phase of fibril growth or the assembly of large oligomers without interfering with early nucleation events [37]. In particular, many natural polyphenols, including resveratrol [38], OleA [29], curcumin [39], and EGCG [30], have been reported to inhibit the fibrillogenesis process of amyloid precursor proteins and peptides in vitro and, possibly, in vivo. We previously reported that OleA hinders the amyloid aggregation of both wt-TTR- and L55P-TTR [29]. In particular, we observed that OleA significantly reduces solvent exposure of those aromatic residues that become uncovered following tetramer disassembly and the ensuing misfolding of the resulting monomers/dimers. All previous published data were collected under acidic conditions, providing an environment suitable to induce tetrameric TTR destabilization, a condition required to trigger the subsequent amyloid aggregation process.

To better interpret previously published data, in the present study, we sought to investigate the molecular features underlying OleA interaction with the structure of tetrameric TTR under physiological conditions (pH 7.0) for each variant. For the first time, we observed that OleA undergoes fluorescence emission following incubation with TTR. This is not surprising, considering that, even in the case of resveratrol and its main metabolites, the fluorescence spectra of these molecules have been used to assess their ability to interact with TTR [40]. The interaction between OleA and TTR induced two distinct effects on OleA fluorescence: an increased fluorescence intensity and a blue shift, even though these phenomena seemed to occur at different protein concentrations. The emission shift appeared at concentrations of wt-TTR higher than 10^−7^ M and was complete at 40 µM. The signal enhancement became evident above 10^−6^ M and did not reach a plateau at 100 µM, the maximum protein concentration we could reach. A plot displaying the blue shift of OleA fluorescence, assessed as the ratio between the signals at 413 and 428 nm, vs. fluorescence intensity illustrates these observations, suggesting that these events may be the spectroscopic signature of two different processes, which appear in the plot as two distinct linear regions (Supplementary Figure S3). Our biophysical investigation allows the blue shift to be assigned to a binding mode, whereby one OleA molecule occupies the binding site of TTR: at low protein concentration, OleA is free in solution and solvent relaxation induces a red shift in the emission of OleA. Indeed, we could obtain a similar blue shift when OleA was incubated with βCD, a molecule providing a hydrophobic cage. Furthermore, the same blue shift was obtained in hydrophobic solvents and the docking studies corroborate this binding mechanism.

As far as the signal increase at high protein concentrations is concerned, it must be noted that this increase was obtained in the presence of different proteins Furthermore, even M-TTR, although devoid of quaternary structure and thus of the binding pocket, exhibited a significant, yet lower, ability to increase OleA fluorescence. These two pieces of evidence suggest that the increase in fluorescence may be due to a non-specific interaction of OleA at the protein surface, rather than to a specific binding in the TTR binding site. However, the fluorescence increase did not reach a plateau at protein concentrations at which the first binding event was saturated and, consequently, no more OleA molecules could still be available under these conditions. A possible explanation of this result is that one OleA molecule interacts with more than one TTR molecule, although the second interaction displays a lower affinity.

The interaction modes described for the first time in the present work are novel but not unique, considering that several small compounds interact with the TTR surface inducing tetramer stabilization and impairing amyloid fibril formation [28]. Among these molecules, natural flavonoids, such as EGCG, do not bind at the T4 binding site and stabilize the tetrameric structure by a stable interaction at the surface of each TTR monomer [28]. In particular, EGCG binds at the dimer–dimer interface, with a resulting effect similar to that of a cross-linker [41]. Our results indicate that OleA binds both at the main funnel and at the hydrophobic surfaces of TTR. Considering the very low toxicity of EGCG and OleA, it would be possible to suggest, and to investigate, the existence of a synergistic effect between these two, and possibly other, polyphenols; the latter could be an encouraging strategy to increase the stabilizing effects of wild-type and mutant forms of TTR by plant polyphenols. 

Our data suggest that OleA displays a *K_D_* of ~3.0 uM for wt-TTR, a value similar to that reported for other polyphenols, such as curcumin (*K_D_*~2.0 uM [42,43]). These values are higher than those reported for drugs used for TTR-amyloidosis such as Tafamidis (4.4 ± 1.3 nM [44]) and Diflunisal (407 ± 35 nM [44]), but this may be not relevant. In fact, Miller and co-workers reported that the *K*_D_ value is not an essential parameter to define a good molecule able to reduce TTR amyloidogenicity [44]. In their study, they analyzed the *K*_D_ values of different drugs such as AG10, Tafamidis, Diflunisal, and Tolcapone and showed that the efficiency of these drugs does not correlate with their *K*_D_ value; rather, it is related to their binding enthalpy values [44]. These data agree with the energy value reported for OleA obtained with the DOCKING assay, where OleA displayed the highest affinity and interaction energy. A different, yet weak, interpretation is that the surface interaction we identified may play an important role with respect to the inhibition of aggregation. Indeed, we previously showed that OleA alters the aggregation pathway, leading to the formation of off-pathway oligomeric assemblies [29]. An interaction occurring at the protein surface seems more suitable to interfere with intermolecular interactions than a binding event occurring within the protein structure. Consequently, the synergistic effect of the two interaction modes described here may underlie the biological activity of OleA.

## 4. Materials and Methods

### 4.1. Oleuropein Deglycosylation

Oleuropein was purchased from Extrasynthese and deglycosilated by treatment with almond β-glycosidase (EC 3.2.1.21, Fluka, Sigma-Aldrich, St. Louis, MI, USA), as previously described [45]. Briefly, a 10 mM solution of oleuropein in 310 μL of 0.1 M sodium phosphate buffer, pH 7.0, was incubated with 8.9 I.U. of β-glycosidase overnight at room temperature. The reaction mixture was centrifuged at 18,000 rpm for 10 min to precipitate OleA, which was resuspended in dimethylsulfoxide (DMSO) in stocks at 50 mM concentration. The complete oleuropein deglycosylation was confirmed by assaying the glucose released in the supernatant with a Glucose (HK) Assay kit (Sigma-Aldrich, St. Louis, MI, USA). Stocks of OleA were kept frozen protected from light and were used within the same day once opened.

### 4.2. Preparation of TTR Samples

Recombinant wt-TTR- and L55P-TTR were expressed and purified according to Mangione et al. [46]. Lyophilized TTR was dissolved at 1.6 mM in 30 mM sodium phosphate buffer, and pH 7.0. M-TTR was purified as previously reported [12]. In brief, the cleared lysate was loaded on a Q-Sepharose High Performance resin (GE Healthcare) and eluted with a sodium chloride gradient. Then, the protein was further purified by gel filtration with a HiLoad 16/600 Superdex 75 pg (GE). The eluted protein was dialyzed against 20 mM potassium phosphate, 150 mM NaCl buffer at pH 7.3 enriched with 2.0 mM DTT.

### 4.3. Docking Experiments

Ligand interaction with the investigated TTR molecules was investigated by molecular docking with the AutoDock Vina software [47]. The reference 3D structures for the simulation were 4MRB (PDB code, wt human TTR pH 7.5, biological tetrameric assembly, resolution 1.27 Å) [48], 2ROX (PDB code, wt human TTR complexed with T4, 2 Å) [2], and 2B14 (PDB code, L55P human amyloidogenic TTR, resolution 2Å) [48] that were prepared using pymol (The PyMOL Molecular Graphics System, Version 2.0 Schrödinger, LLC.) and AutodockTools (ADT) from MGLTools v. 1.5.6 RC3. For 2ROX, the T4 ligand was removed by manually editing the PDB file and eliminating the lines concerning the T4 molecule.

The structures of the ligands were taken from the ZINC database [49]. In addition to oleuropein (id 4098348), T4 (thyroxine, id 3830993) and resveratrol (id 6787) were also obtained. The Zinc database structure editor was used to design OleA by removing the glucose moiety from native oleuropein. Then, both protein and ligands were prepared for docking in ADT by adding explicit hydrogen atoms and saving in pdbqt format. For ligands, all possible torsions were enabled.

Docking grids were designed in ADT using as a reference the TTR tetrameric structure coordinates to locate (1) a large area around the tetrameric protein; (2) the crystal-resolved docking site of T4 (main funnel); and (3) the halogen binding sites (secondary funnel). All the files used for docking are available at https://github.com/matteoramazzotti/papers/2021ttr_dock (accessed on 20 January 2022).

### 4.4. Interaction between OleA and wt-TTR

We prepared 21 samples containing OleA at a concentration of 500 nM and wt-TTR at concentrations in the 10^−8^–10^−4^ M range in 20 mM phosphate buffer, pH 7.4, in a total volume of 60 µL. The fluorescence of each sample was recorded using an Agilent Cary Eclipse spectrofluorometer (Agilent Technologies, Santa Clara, CA, USA) equipped with a thermostated cell holder attached to an Agilent PCB 1500 water Peltier system, using a low-volume quartz cuvette (Hellma, Müllheim, Germany). The excitation wavelength was 340 nm, and the fluorescence spectra were collected in the 400 nm–600 nm range. We used excitation and emission slits of 5.0 nm, a PMF signal of 860 V, and a temperature of 25 °C. To analyze the obtained data, the fluorescence of the blank solution was subtracted from each spectrum, and the total fluorescence emitted at each wt-TTR concentration was calculated as the sum of all the fluorescence values of each spectrum. Then, the spectra obtained (Figure 3a) were normalized to the signal emitted at 420 nm (Figure 3b) to highlight possible emission shifts. Last, for each spectrum, the peak wavelength ratio between the emission at 428 nm and 413 nm and the center of emission (COM) were calculated according to the equation $\mathrm{COM}=\underset{i}{\sum }F_{i}/\underset{i}{\sum }\left(F_{i}\cdot {\bar{\nu }}_{i}\right)$, where $F_{i}$ is the signal emitted at a wavenumber ${\bar{\nu }}_{i}$. The plot of COM and peak wavelength vs. [wt-TTR] was normalized to the fraction of OleA bound to wt-TTR (Figure 3d) (bf), and the plot obtained was analyzed with a binding equilibrium of the type TTR + OleA ⇌ TTR∙OleA, where TTR∙OleA denotes the complex between the two molecules. The trace obtained was fitted to an equation

$$\mathrm{bf}\;=\;1/\{1\;+\;K_{D}/[\mathrm{TTR}]\}$$

where $K_{D}$ is the dissociation constant. The same experiment was repeated with L55P-TTR and M-TTR.

### 4.5. Biophysical Characterization of OleA Emission

In a first experiment, we prepared two samples with a total volume of 60 µL containing OleA at a concentration of 200 µM, in the presence or in the absence of 200 µM β-cyclodextrin (Sigma-Aldrich, St. Louis, MI, USA). The fluorescence was recorded with the same instrument and cuvette as indicated above. The excitation wavelength was 350 nm; the excitation and emission slits were 5.0 and 10 nm. The traces reported in Figure 3 are the average of 32 scans at 900 V.

In a second set of experiments, the fluorescence spectra of samples containing OleA at a concentration of 200 µM, dissolved in different solvents, in a total volume of 60 µL were acquired. The solvents (all from Sigma-Aldrich, St. Louis, MI, USA) were water (ε/ε_0_ = 80.0), methanol (ε/ε_0_ = 32.7), ethanol (ε/ε_0_ = 24.5), 1-propanol (ε/ε_0_ = 21.8), 1-butanol (ε/ε_0_ = 17.5), 1-heptanol (ε/ε_0_ = 12.1), and 1-octanol (ε/ε_0_ = 10.3). The excitation wavelength was 340 nm; excitation and emission slits were 5.0 nm. The spectra shown in the inset of Figure 5b are the average of 16 scans at 950 V. Finally, the fluorescence emitted at 485 nm was measured in samples containing OleA at a concentration of 25 µM, dissolved in water and in the presence of 100 µM wt-TTR, 100 µM bovine serum albumin (BSA, Sigma, St. Louis, MI, USA), or hen egg white lysozyme (HEWL, Sigma, St. Louis, MI, USA). The excitation wavelength was 340 nm; excitation and emission slits were 5.0 nm. The obtained fluorescence values were normalized to the signal recorded in the absence of protein. Each of the values reported in Figure 3c is the average of 5 readings. Error bars are SEM.

### 4.6. Intrinsic Fluorescence Measurements

Spectra of wt-TTR, L55P-TTR, and M-TTR intrinsic fluorescence were collected at 295 nm excitation before and after OleA supplementation; the emission intensity was scanned in the 300–400 nm range. The fluorescence emission spectra were normalized to an intensity of 1.0 at the observed peak wavelength using the FL WinLab software (Perkin-Elmer Instrument Corporation, Wellesley, MA, USA) prior to derivatization. Normalization of aromatic residue emission scans was essential to compare the intensities and the positions of the various bands appearing in the second derivatives of the fluorescence emission scans. Five independent spectra were averaged, smoothed with a 15-sliding point sliding window average and a Savitzky–Golay algorithm, and finally, the second derivatives of the smoothed spectra were obtained using Omnic-software (Nicolet Inc., Madison, WI, USA) [50]. Such a smoothing step was needed to reduce the noise in the second derivative and designed to meet two main criteria: (i) the overall shape and intensity of the raw emission scan was not affected by the smoothing and (ii) the overall shape of the bands in the second derivative was preserved.

### 4.7. Fluorimetric Binding Assays

Excitation–emission matrices (EEM) were performed in the 280–340 nm excitation range and in the 350–600 nm emission range. The excitation spectra were determined by measuring the emission intensity at a fixed wavelength of 440 nm while varying the excitation wavelength in the 300–400 nm range (5.0 nm by 5.0 nm). For the emission spectra, the excitation was at 360 nm and the emission was monitored in the 380–550 nm range. Fluorescence binding experiments were carried out in 50 mM sodium phosphate buffer, 150 mM sodium chloride, pH 7.4, at 37 °C, using a Perkin Elmer LS-50B spectrofluorometer.

## 5. Conclusions

In conclusion, our work provides a combined strategy of molecular docking and fluorescence assays to explore the molecular basis of the mechanism of TTR-OleA binding, with ensuing stabilization of the protein native structure. In addition, a better knowledge of this interaction at the molecular level offers a crucial basis for the use of OleA as a possible preventive and therapeutic agent against various types of TTR amyloidosis. Moreover, protein–polyphenol binding can modify the property of the polyphenol itself, stabilizing the compound and increasing its bioavailability and bioactive functions such as its antioxidant activity [51]. Overall, this study suggests that OleA merits consideration for the development of preventives and therapeutics against TTR amyloidosis, contributing to support the importance of a healthy lifestyle, including safe nutrition such as that provided by the Mediterranean diet, rich in plant polyphenols.

## Data Availability

Data is contained within the article and Supplementary Materials.

## Supplementary Materials

The following are available online at https://www.mdpi.com/article/10.3390/ph15030277/s1, Figure S1: Calculation of the center of mass (COM) and the ratio between the emission at 428 nm and 413 nm of OleA as a function of wt-TTR concentration. Figure S2: Comparison of the emission spectra of OleA in the presence of wt-TTR, L55P-TTR and M-TTR. Figure S3: A phase diagram showing the blue shift of OleA fluorescence upon increasing wt-TTR concentration.
