# Supplementary material for: The Transthyretin/Oleuropein Aglycone Complex: A New Tool against TTR Amyloidosis
Source: Pharmaceuticals (Basel). 2022 Feb 23;15(3):277. doi: 10.3390/ph15030277 (PMC8953266; doi:10.3390/ph15030277)
Supplement: Supplementary file 1 [file pharmaceuticals-15-00277-s001.zip › pharmaceuticals-1592554-supplementary.pdf]

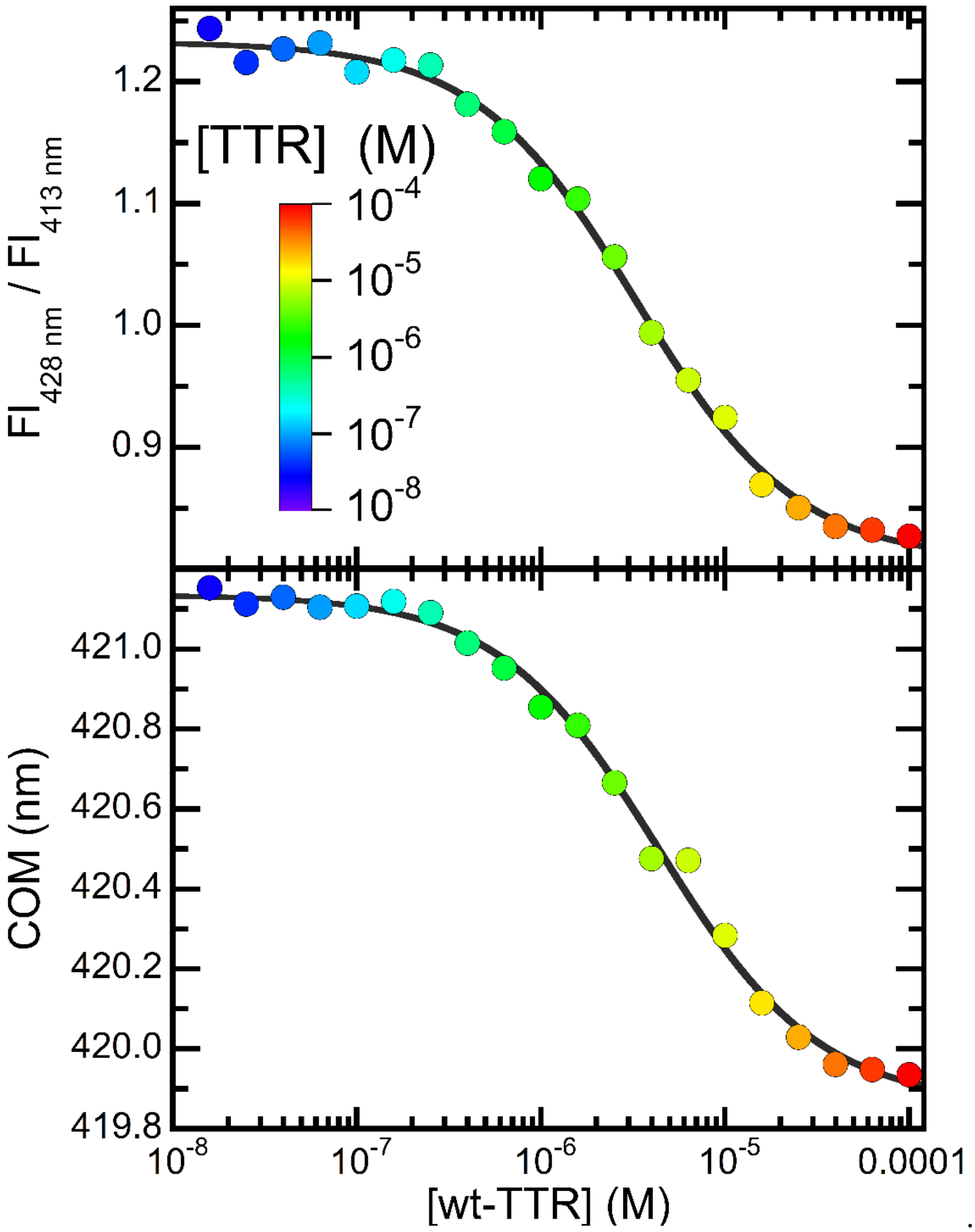

Figure S1. Calculation of the center of mass (COM, bottom panel) and the ratio between the emission at 428 nm and 413 nm (top panel) of OleA as a function of wt-TTR concentration. The continuous lines represent best fits of experimental data to the binding equation reported in the methods section. The color refers to protein concentration according to the scale reported in the top panel.

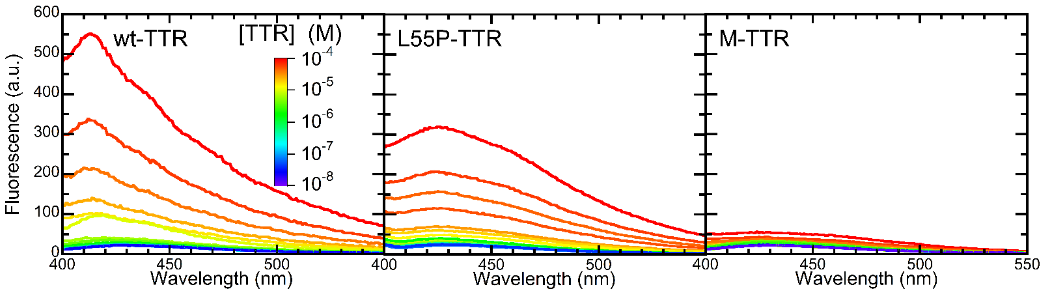

Figure S2. Comparison of the emission spectra of OleA in the presence of wt-TTR (left panel, the same spectra are shown in Fig. 1A, in the main text), L55P-TTR (central panel) and M-TTR (right panel). The spectra are shown on the same scale and were collected using the same instrumental settings. The color refers to protein concentration according to the scale reported in the left panel.

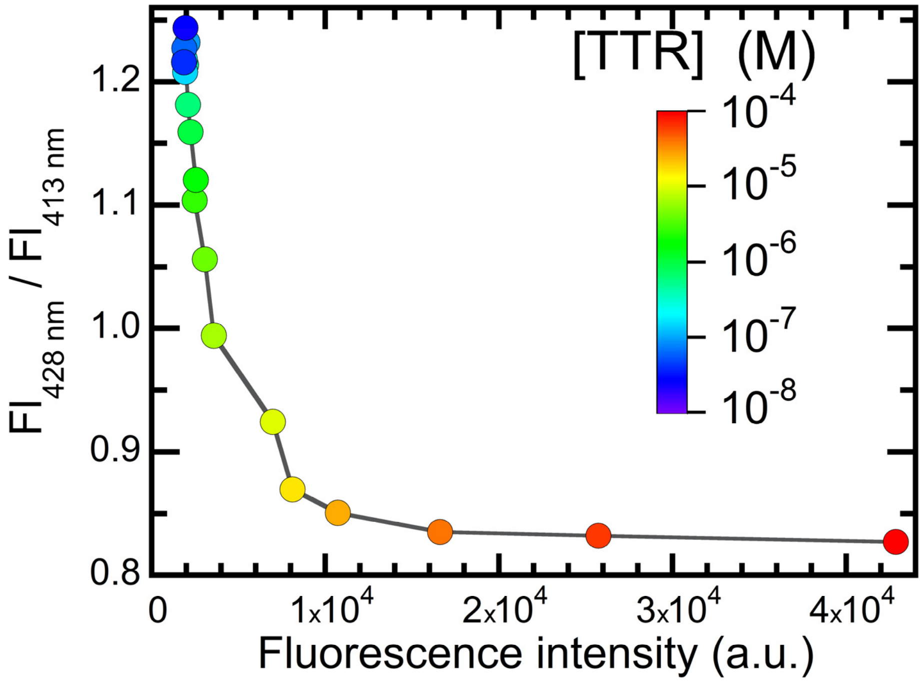

.

Figure S3. A phase diagram showing the blue shift of OleA fluorescence upon increasing wt-TTR concentration, reported on the *y*-axis as the ratio between the fluorescence signals emitted at 428 nm and 413 nm, vs. OleA fluorescence intensity, reported on the *x*-axis. The experimental points are connected by a continuous line, to highlight the observed trend on increasing TTR concentration. The two linear regions, corresponding roughly to the TTR concentration intervals of 1×10^{-8}–10^{-5} M and 1×10^{-5}–10^{-4} M, illustrate two distinct interaction modes between the two molecules.
